# Turmeric Root and Its Bioactive Ingredient Curcumin Effectively Neutralize SARS-CoV-2 In Vitro

**DOI:** 10.3390/v13101914

**Published:** 2021-09-23

**Authors:** Maren Bormann, Mira Alt, Leonie Schipper, Lukas van de Sand, Vu Thuy Khanh Le-Trilling, Lydia Rink, Natalie Heinen, Rabea Julia Madel, Mona Otte, Korbinian Wuensch, Christiane Silke Heilingloh, Thorsten Mueller, Ulf Dittmer, Carina Elsner, Stephanie Pfaender, Mirko Trilling, Oliver Witzke, Adalbert Krawczyk

**Affiliations:** 1 Department of Infectious Diseases, West German Centre of Infectious Diseases, Universitätsmedizin Essen, University Duisburg-Essen, 45147 Essen, Germany; maren.bormann@uk-essen.de (M.B.); Mira.Alt@uk-essen.de (M.A.); Leonie.Schipper@uk-essen.de (L.S.); Lukas.vandeSand@uk-essen.de (L.v.d.S.); Rabea.Madel@uk-essen.de (R.J.M.); Mona.Otte@uk-essen.de (M.O.); Korbinian.Wuensch@uk-essen.de (K.W.); Christiane.Heilingloh@uk-essen.de (C.S.H.); Oliver.Witzke@uk-essen.de (O.W.); 2 Institute for Virology, University Hospital Essen, University Duisburg-Essen, 45147 Essen, Germany; Khanh.Le@uk-essen.de (V.T.K.L.-T.); Lydia.Rink@uk-essen.de (L.R.); Ulf.Dittmer@uk-essen.de (U.D.); Carina.Elsner@uk-essen.de (C.E.); Mirko.Trilling@uk-essen.de (M.T.); 3 Department of Molecular and Medical Virology, Faculty of Medicine, Ruhr University Bochum, 44801 Bochum, Germany; Natalie.Heinen@ruhr-uni-bochum.de (N.H.); stephanie.pfaender@ruhr-uni-bochum.de (S.P.); 4 Department of Molecular Biochemistry, Cell Signaling, Ruhr-University Bochum, 44801 Bochum, Germany; thorsten.t.mueller@rub.de; 5 Institute of Psychiatric Phenomics and Genomics (IPPG), University Hospital, LMU Munich, 80336 Munich, Germany

**Keywords:** SARS-CoV-2, COVID-19, herbal medicine, antiviral, *Curcuma longa*, turmeric root, curcumin

## Abstract

Severe Acute Respiratory Syndrome Coronavirus Type 2 (SARS-CoV-2) is the causative agent of the coronavirus disease 2019 (COVID-19). The availability of effective and well-tolerated antiviral drugs for the treatment of COVID-19 patients is still very limited. Traditional herbal medicines elicit antiviral activity against various viruses and might therefore represent a promising option for the complementary treatment of COVID-19 patients. The application of turmeric root in herbal medicine has a very long history. Its bioactive ingredient curcumin shows a broad-spectrum antimicrobial activity. In the present study, we investigated the antiviral activity of aqueous turmeric root extract, the dissolved content of a curcumin-containing nutritional supplement capsule, and pure curcumin against SARS-CoV-2. Turmeric root extract, dissolved turmeric capsule content, and pure curcumin effectively neutralized SARS-CoV-2 at subtoxic concentrations in Vero E6 and human Calu-3 cells. Furthermore, curcumin treatment significantly reduced SARS-CoV-2 RNA levels in cell culture supernatants. Our data uncover curcumin as a promising compound for complementary COVID-19 treatment. Curcumin concentrations contained in turmeric root or capsules used as nutritional supplements completely neutralized SARS-CoV-2 in vitro. Our data argue in favor of appropriate and carefully monitored clinical studies that vigorously test the effectiveness of complementary treatment of COVID-19 patients with curcumin-containing products.

## 1. Introduction

In early 2020, Severe Acute Respiratory Syndrome Coronavirus Type 2 (SARS-CoV-2) was identified as the causative agent of the coronavirus disease 2019 (COVID-19) that is currently causing a global pandemic [1]. The major transmission routes of SARS-CoV-2 are droplet and airborne transmission [2]. SARS-CoV-2 infections can be asymptomatic or cause a respiratory and other severe diseases. Symptoms range from mild, cold-like symptoms including fever and cough to severe, life-threatening disease [3].

The current therapeutic treatment options for COVID-19 comprise antiviral therapeutics such as remdesivir as well as immunomodulatory therapeutics such as dexamethasone to downregulate hyper-inflammatory immune responses [4,5]. However, the availability of potent antiviral compounds is limited, particularly in developing areas, and the development of novel antiviral compounds is time- and cost-intensive and may take years before approval. Traditional herbal medicines represent promising options for complementary treatment of COVID-19 diseases. To date, numerous plants and their ingredients exhibit potent antimicrobial and antiviral effects [6,7,8]. Notably, curcumin showed antimicrobial activity toward bacteria, malaria, fungi, and viruses [9].

Turmeric root, also known as *Curcuma longa*, is broadly used as a spice widely cultivated in Southeast Asia. The rhizome of *Curcuma longa* contains several structurally related curcuminoids. Sixty to 75% of the curcuminoid content consists of curcumin, also known as diferuloylmethane. The remaining fraction is a combination of demethoxycurcumin (20–25%) and bisdemethoxycurcumin (5–15%) [10,11]. Turmeric root has been used for thousands of years as medicine for the complementary treatment of a wide variety of diseases. As early as 1815, the bioactive ingredient curcumin was first isolated from turmeric root by Vogel and Pelletier. Curcumin reveals a broad spectrum of bioactivities such as antioxidant, anti-inflammatory, antibacterial, antiviral, antitumor, and hepatoprotective activities [12,13,14,15].

We and others have demonstrated the antiviral activities of curcumin against various viruses, including Dengue Virus, Human Immunodeficiency Virus (HIV), Kaposi Sarcoma-associated Herpesvirus, Enterovirus, Zika Virus, Chikungunya Virus, Vesicular Stomatitis Virus, the Human Respiratory Syncytial Virus, Viral Hemorrhagic Septicemia Virus, Influenza A Virus, Herpes Simplex Type 2, Norovirus, and Hepatitis C Virus [16,17]. Furthermore, curcumin is known for its pharmacological abilities especially as an anti-inflammatory and antiviral agent [18,19]. Moreover, curcumin was discussed as a potential candidate in the therapeutic regimen of COVID-19 [20]. However, its antiviral activity against SARS-CoV-2 has, at least to our knowledge, not been thoroughly proven so far. There is clearly a medical need to determine if curcumin may have a direct antiviral activity and thus may be suitable for complementary treatment of COVID-19.

In the present study, we investigated the neutralizing activity of aqueous turmeric root extract, curcumin-containing nutritional supplement capsules, and pure curcumin against SARS-CoV-2.

## 2. Materials and Methods

### 2.1. Aqueous Turmeric Root Extract

Turmeric root was comminuted through a grater and subsequently centrifuged (10 min, 3985 RCF (relative centrifugal force)) to remove solid components. The supernatant was further purified by ultracentrifugation at 50,624 RCF and 4 °C for two hours.

### 2.2. Curcumin-Containing Nutritional Supplement Capsule

One curcumin-containing nutritional supplement capsule (Nature Love, Tauron Ventures GmbH, Monheim am Rhein, Germany) was dissolved in 10 mL of dimethyl sulfoxide (DMSO) (Roth, Karlsruhe, Germany). The solution was subsequently diluted 1:10 (*v*/*v*) in Dulbecco’s modified Eagle’s medium (DMEM), containing 10% (*v*/*v*) fetal calf serum (FCS), penicillin (100 IU/mL), and streptomycin (100 µg/mL) (all Life Technologies Gibco, Darmstadt, Germany) and stored at 37 °C. One capsule contained 640 mg turmeric powder, 105 mg turmeric extract (containing 99.9 mg curcumin), and 5 mg black pepper (containing 4.7 mg piperine).

### 2.3. Curcumin

For cell culture experiments, 200 mg of curcumin (diferuloylmethane) (Sigma Aldrich, Darmstadt, Germany) was dissolved in 10 mL DMSO and subsequently diluted 1:10 (*v*/*v*) in DMEM, containing 10% (*v*/*v*) FCS, penicillin (100 IU/mL), and streptomycin (100 µg/mL) and stored at 37 °C.

### 2.4. Cells and Virus

Vero E6 cells (African green monkey kidney cells, American Type Culture Collection, ATCC, Manassas, Virginia, USA; ATCC^®^ CRL-1586™) were cultivated in DMEM, containing 10% (*v*/*v*) FCS, penicillin (100 IU/mL), and streptomycin (100 µg/mL) at 5% CO_2_ and 37 °C. Calu-3 cells (human lung cancer cell line, American Type Culture Collection, ATCC, Manassas, Virginia, USA; ATCC^®^ HTB-55™) were cultivated in Eagle’s minimum essential medium (EMEM; ATCC, Manassas, Virginia), containing 10% (*v*/*v*) FCS, penicillin (100 IU/mL), and streptomycin (100 µg/mL) at 5% CO_2_ and 37 °C. The clinical SARS-CoV-2 isolate was derived from a nasopharyngeal swab of a patient with COVID-19 hospitalized in April 2020 at the University Hospital in Essen as previously described [21]. The virus was propagated on Vero E6 cells maintained in DMEM supplemented with 10% (*v*/*v*) FCS, penicillin (100 IU/mL), streptomycin (100 µg/mL), ciprofloxacin (10 µg/mL), and amphotericin B (2.5 µg/mL). After five days of incubation, the supernatant was harvested, cleared from cell debris by centrifugation, and stored at −80 °C. Viral titers were determined by a standard endpoint dilution assay and calculated as 50% tissue culture infective dose (TCID_50_)/mL as previously described [22].

### 2.5. Neutralization Assay on Vero E6 Cells

The neutralization capacity of aqueous turmeric root extract, curcumin-containing nutritional supplement capsules, and pure curcumin were determined in cell culture by endpoint dilution as described previously [23]. For this purpose, serial twofold dilutions of turmeric root extract (1:8–1:1024), nutritional supplement capsules (468.8–3.7 µg/mL), and curcumin (125–1 µg/mL) were pre-incubated with 100 TCID_50_ of SARS-CoV-2 for one hour at 37 °C and subsequently incubated on confluent Vero E6 cells grown on 96-well microtiter plates. Untreated Vero E6 cells served as negative control and cells infected with 100 TCID_50_ of SARS-CoV-2 in the absence of antiviral compounds served as positive control. After 48 h, cells were stained with 0.5% crystal violet (*w*/*v*) (Roth, Karlsruhe, Germany), solved in 20% (*v*/*v*) methanol (Merck, Darmstadt, Germany), and analyzed for cytopathic effects (CPE) by transmitted light microscopy (Carl Zeiss AG, Oberkochen, Germany). The concentration required for reducing virus-induced CPE by 100% was determined as the complete neutralization titer. To determine the half-maximal effective concentration (EC_50_) sufficient to neutralize the virus, for each dilution, the percentage of cell cultures showing CPEs was determined. EC_50_ values were calculated by nonlinear regression, and the means were calculated using GraphPad Prism 8.0.1. The experiment was performed three times independently.

### 2.6. Neutralization Assay via icELISA on Human Calu-3 Cells

The neutralization efficacy of curcumin against SARS-CoV-2 on human Calu-3 cells was assessed by an in-cell ELISA (icELISA)-based neutralization test (icNT). The icNT was performed as described previously [24]. In brief, 5000 plaque-forming units (PFU) of SARS-CoV-2 were incubated with different dilutions of turmeric root extract (1:16–1:128), nutritional supplement capsule content (468.8–58.6 µg/mL), or curcumin (125–15.6 µg/mL) for 1 h prior to inoculation of Calu-3 cells seeded on 96-well plates (≈5 × 10^4^ cells per well). At 24 h post infection, Calu-3 cells were fixed with 4% (*w*/*v*) paraformaldehyde/PBS, permeabilized with 1% (*v*/*v*) Triton-X-100/PBS, and blocked with 3% (*v*/*v*) FCS/PBS. An SARS-CoV-2 N-specific primary antibody was added and incubated overnight at 4 °C. The cells were washed three times with 0.05% (*v*/*v*) Tween-20/PBS followed by incubation with a peroxidase-labeled secondary antibody for 1 h. After four washing steps, the enzyme reaction was visualized by adding tetramethylbenzidine (TMB) substrate and stopped with 0.5 M HCL. The absorbance was measured with a microplate multireader at OD 450 (Mithras2 LB 943; Berthold Technologies). Means and standard errors of the mean were calculated and significance was assessed by a one-way analysis of variance (ANOVA) and Dunnett’s multiple comparison test using GraphPad Prism 8.0.1.

### 2.7. Cell Viability Assay

A potential cytotoxicity of various concentrations of aqueous turmeric root extract, nutritional supplement capsules, and curcumin toward Vero E6 cells was determined using the Orangu cell-counting solution (Cell guidance systems, Cambridge, UK) as described before [7]. Orangu™ is a colorimetric assay used in cytotoxicity assays for the calculation of viable cell numbers. In this assay, WST-8 tetrazolium salt is reduced by cellular dehydrogenase activities to an orange formazan product. The quantity of living cells is directly proportional to the amount of chemically converted orange-colored formazan dye [25]. Orangu assay was conducted according to the manufacturer’s instructions. Identical twofold dilutions of aqueous turmeric root extract, nutritional supplement capsules, and curcumin as used in the neutralization assays and icNTs were utilized to overlay 96-well microtiter plates, which contained Vero E6 or Calu-3 cells. The further incubation took place at 37 °C and 5% CO_2_ according to the duration of the corresponding neutralization test for 48 h (Vero E6 cells) or 24 h (Calu-3 cells). After incubation, 10 µL of Orangu cell counting solution was added to the wells and incubated for 120 min (37 °C, 5% CO_2_). Cell viability was measured at an absorbance of 450 nm using Mithras LB 940 (Berthold Technologies, Oak Ridge, TN, USA). The experiment was performed three times independently. The means and standard error of the mean were calculated using GraphPad Prism 8.0.1 (Graph Pad Software, San Diego, CA, USA).

### 2.8. Quantitative SARS-CoV-2 RT-PCR

We assessed the effect of aqueous turmeric root extract, nutritional supplement capsules, and curcumin on SARS-CoV-2 RNA levels by qRT-PCR. In brief, we co-incubated 100 TCID_50_ SARS-CoV-2 with serial dilutions of curcumin (125–1 µg/mL) at 37 °C for one hour. Virus–curcumin suspensions were added to confluent Vero E6 cells grown in 96-well plates. Untreated Vero E6 cells and cells treated with 100 TCID_50_ SARS-CoV-2 served as controls. Subsequently, supernatants were harvested, and the viral RNA was purified using the QIAamp Viral RNA Mini Kit (QIAGEN, Hilden, Germany). The genomic SARS-CoV-2 RNA was quantified by RT-qPCR, using primers targeting the viral M or N gene [26]. Plasmid dilution series of 1:10 were used as reference to assess the M and N gene copy numbers (details and sequence information available upon request). The experiment was performed three times independently. EC_50_ were calculated by nonlinear regression, and the means were calculated using GraphPad Prism 8.0.1.

## 3. Results

### 3.1. In Vitro Neutralization of SARS-CoV-2

The turmeric root ingredient curcumin plays an important role in traditional medicine because of its anti-inflammatory and antimicrobial activity. Here, we investigated the antiviral effect of an aqueous turmeric root extract, curcumin-containing nutritional supplement capsules, and pure curcumin against SARS-CoV-2.

**Figure 1:**
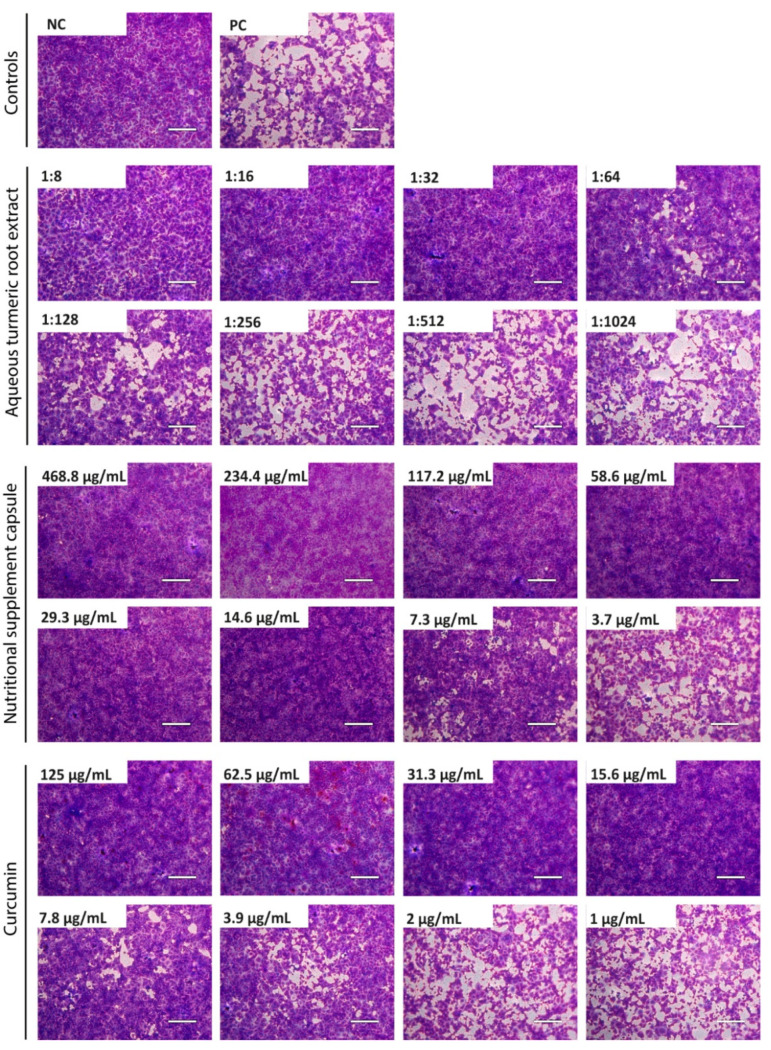
Neutralization of SARS-CoV-2 by aqueous turmeric root extract, curcumin-containing nutritional supplement capsules, and curcumin. Decreasing concentrations of aqueous turmeric root extract (1:8–1:1024 dilution), nutritional supplement capsules (468.8–3.7 µg/mL), and curcumin (125–1 µg/mL) were pre-incubated with 100 TCID_50_ of SARS-CoV-2 for one hour and subsequently added to confluent Vero E6 cells. After 48 h, cells were stained with crystal violet and analyzed for cytopathic effects using transmitted light microscopy. The experiment was performed three times independently. Representative images are displayed. NC = negative control (medium); PC = positive control (100 TCID_50_ SARS-CoV-2); scale bar = 200 µm.

**Figure 2:**
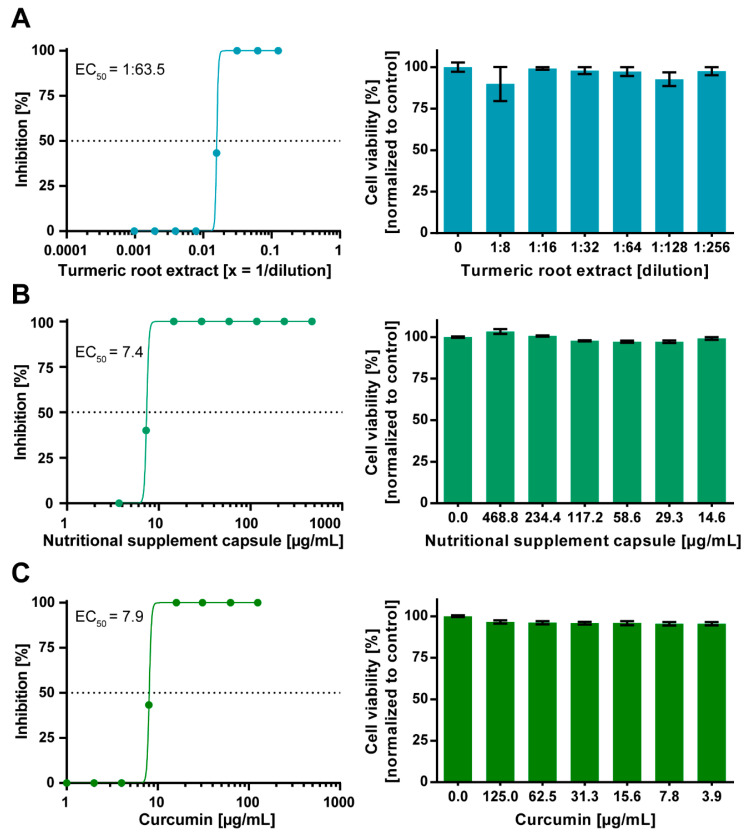
Dose-dependent antiviral activity of aqueous turmeric root extract, curcumin-containing nutritional supplement capsules, and curcumin against SARS-CoV-2. Decreasing concentrations of aqueous turmeric root extract (1:8–1:1024 dilution) (**A**), nutritional supplement capsules (468.8–3.7 µg/mL) (**B**), and curcumin (125–1 µg/mL) (**C**) were pre-incubated with 100 TCID_50_ of SARS-CoV-2 for one hour. Subsequently, each dilution of virus–herb suspensions was incubated on confluent Vero E6 cells grown on a 96-well plate. After 48 h, cells were stained with crystal violet and analyzed regarding cytopathic effects. The half-maximal effective concentration (EC_50_) was calculated by nonlinear regression using GraphPad Prism. The cytotoxic effect of various concentrations of aqueous turmeric root extract, nutritional supplement capsules, and curcumin toward Vero E6 cells was determined by Orangu Cell Counting Solution (Cell guidance systems) after 48 h. Cell viability was normalized to untreated control cells. The experiment was performed three times independently. Error bars represent the standard error of the mean (SEM).

Various concentrations of turmeric root extract (1:8–1:1024 dilution), curcumin-containing nutritional supplement capsules (468.8–3.7 µg/mL), and pure curcumin (125–1 µg/mL) dissolved in cell-culture medium were incubated with 100 TCID_50_ of SARS-CoV-2 for 1 h. Subsequently, mixtures were incubated on confluent Vero E6 cells. Two days after infection, cell cultures were fixed and stained with crystal violet and microscopically inspected for a CPE. The concentration required for complete virus neutralization (Figure 1) was determined, and EC_50_ values were calculated (Figure 2).

Complete neutralization of 100 TCID_50_ SARS-CoV-2 was achieved by aqueous turmeric root extract at a dilution of 1:32 (Figure 1). In order to calculate the EC_50_ of turmeric root extract, we quantitatively analyzed the neutralization assay. Dose–response assessment showed that the EC_50_ of turmeric root extract was achieved at a dilution of 1:63.5 (Figure 2A). No cytotoxic effect for turmeric root extract was observed at the indicated dilutions (Figure 2A). The dissolved nutritional supplement capsules completely neutralized SARS-CoV-2 at a concentration of 14.6 µg/mL (Figure 1). The EC_50_ was determined at a concentration of 7.4 µg/mL (Figure 2B). No cytotoxic effect for the nutritional supplement capsule was observed (Figure 2B). Curcumin is the main bioactive component of turmeric root and was already shown to have an elicit antiviral activity against various viruses [11,16]. Curcumin achieved the complete neutralization of SARS-CoV-2 until a subtoxic concentration of 15.6 µg/mL (Figure 1) with an EC_50_ of 7.9 µg/mL (Figure 2C). Effects related to cytotoxicity were excluded, since none of the applied concentrations exhibited cytotoxicity (Figure 2C).

**Figure 3:**
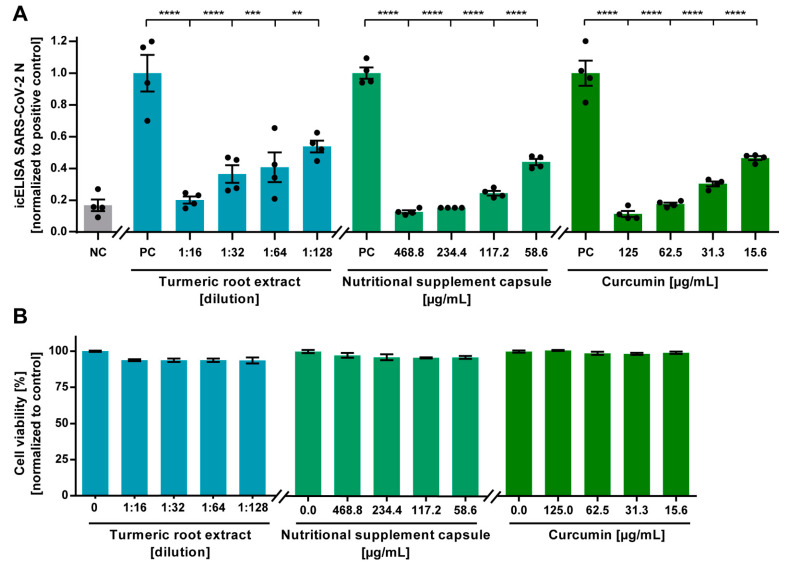
Neutralization of SARS-CoV-2 by aqueous turmeric root extract, curcumin-containing nutritional supplement capsules, and curcumin on a human cell line assessed by an in-cell ELISA (icELISA)-based neutralization test (icNT). (**A**) Decreasing concentrations of aqueous turmeric root extract (1:16–1:128 dilution), nutritional supplement capsule content (468.8–58.6 µg/mL), or curcumin (125–15.6 µg/mL) were pre-incubated with 5000 plaque-forming units (PFU) of SARS-CoV-2 for one hour. Subsequently, mixtures were added to human Calu-3 cells and incubated for 24 h. After incubation with a SARS-CoV-2 N-specific primary antibody and peroxidase-labelled secondary antibody, the enzyme reaction was visualized by adding tetramethylbenzidine. Absorbance was measured with a microplate multireader at OD450. Statistical analysis was performed with one-way analysis of variance (ANOVA) and Dunnett’s multiple comparison test using GraphPad Prism. ** *p* < 0.01; *** *p* < 0.001; and **** *p* < 0.0001; error bars represent the standard error of the mean (SEM). NC = negative control (medium); PC = positive control (5000 PFU SARS-CoV-2). (**B**) The cytotoxic effect of various concentrations of aqueous turmeric root extract, nutritional supplement capsule, and curcumin toward Calu-3 cells was determined by Orangu™ Cell Counting Solution (Cell guidance systems) after 24 h. The cell viability was normalized to untreated control cells. Error bars represent the SEM.

To assess the antiviral effect of turmeric root extract, dissolved nutritional supplement capsules, and curcumin against SARS-CoV-2 on human cells, we conducted an icNT on Calu-3 cells. Different compound dilutions were incubated with 5000 PFU of SARS-CoV-2 for 1 h prior to inoculation of Calu-3 cells. After 24 h, the extent of the infection was quantified after staining of the SARS-CoV-2 N antigen by icELISA. All tested mixtures potently neutralized SARS-CoV-2 at subtoxic concentrations (Figure 3A). Low concentrations of 58.6 µg/mL of the dissolved supplement capsules, 15.6 µg/mL of curcumin, and a high dilution of 1:128 of turmeric root extract were sufficient to significantly neutralize SARS-CoV-2. No cytotoxic effect could be observed at the indicated concentrations (Figure 3B).

In conclusion, we demonstrated, to our knowledge, for the first time a potent antiviral activity of turmeric root and its bioactive ingredient curcumin against SARS-CoV-2 on Vero-E6 and Calu-3 cells.

### 3.2. Effect of Curcumin on SARS-CoV-2 RNA

Curcumin was described as the main active ingredient of turmeric root. Therefore, we further tested whether curcumin may have an effect on RNA levels of SARS-CoV-2 in cell culture. For this purpose, serial dilutions of curcumin were co-incubated with 100 TCID_50_ SARS-CoV-2 for 1 h prior to inoculation of Vero E6 cells. Two days post infection, supernatants were harvested, and SARS-CoV-2 RNA was quantified by RT-qPCR.

**Figure 4:**
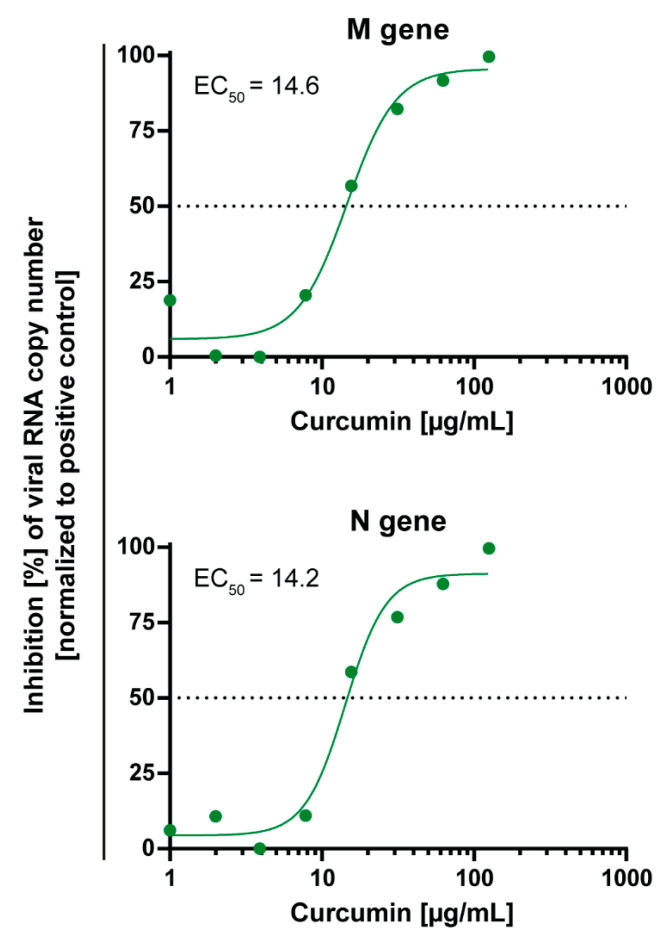
Dose-dependent activity of curcumin on SARS-CoV-2 RNA genome copy numbers. Decreasing concentrations of curcumin (125–1 µg/mL) were pre-incubated with 100 TCID_50_ of SARS-CoV-2 for one hour and subsequently added to confluent Vero E6 cells. After 48 h, cell culture supernatants were harvested, and the genomic SARS-CoV-2 RNA was quantified via RT-qPCR, using primer targeting the viral M or N gene. Cells infected with 100 TCID_50_ of SARS-CoV-2 served as positive control. The experiment was performed three times independently. The half-maximal effective concentration (EC_50_) was calculated by nonlinear regression using GraphPad Prism.

The RT-qPCR results correlated very well with the aforementioned experiments assessing the neutralization efficacy by CPE inspection. Curcumin significantly reduced SARS-CoV-2 RNA levels in cell culture supernatants with an EC_50_ of ≈14 µg/mL (Figure 4).

## 4. Discussion

Herbal medicines with antiviral activity are promising candidates for complementary treatment of viral infections such as SARS-CoV-2 infections since they are cost-effective and broadly available around the world. In the present study, we showed that turmeric root and its bioactive ingredient curcumin have a strong antiviral effect against SARS-CoV-2 in cell culture at low subtoxic concentrations. These findings highlight curcumin as an antiviral compound against SARS-CoV-2.

Using natural products or repurposing drugs to develop antiviral agents can be an alternative strategy to the time-consuming process of developing or designing new compounds. Turmeric root has a long history as a medicine for a variety of uses around the world, including as an antiseptic, anti-inflammatory agent with antimicrobial activity [27]. Aqueous turmeric root extract, dissolved nutritional supplement capsules, as well as curcumin potently neutralized SARS-CoV-2 in Vero E6 and Calu-3 cell culture models. Furthermore, curcumin significantly reduced SARS-CoV-2 RNA levels in cell culture supernatants. A possible mechanism of action may be the inhibition of viral entry by curcumin. Former in silico studies indicated that curcumin may interfere with the binding of the spike (S) glycoprotein of SARS-CoV-2 to angiotensin-converting enzyme 2 (ACE-2) receptor [28,29]. The ACE-2 receptor is located on the surface of several cell types in humans, including secretory goblet cells in the nasal mucosa, absorptive enterocytes in the intestine, as well as type II pneumocytes in the lung [30]. In silico, the keto and enol forms of curcumin established potent hydrogen bonding with the ACE-2 receptor [28]. Recently, another in silico study predicted that curcumin strongly binds to the receptor-binding domain (RBD) of the S-protein, the ACE-2 receptor, and the complex between the RBD and ACE-2 [29].

Curcumin acts as an antiviral agent against a variety of viruses, including HIV, HCV, Influenza A, and Severe Acute Respiratory Syndrome Coronavirus 1 (SARS-CoV-1) [16,17,31]. To inhibit the integrase of HIV-1, an EC_50_ of 40 µM curcumin is necessary compared to the effective dose of 10 µM against influenza A virus [16]. Furthermore, curcumin inhibits SARS-CoV-1 with an EC_50_ of >10 µM [31]. We showed that curcumin also inhibits SARS-CoV-2 CPEs with an EC_50_ of 7.8 µg/mL (21.2 µM) of infected Vero E6 cells, which is a standard in vitro model in SARS-CoV-2 research. Furthermore, curcumin treatment significantly reduced SARS-CoV-2 RNA levels in cell culture supernatants of Vero E6 cells with an EC_50_ of ≈14 µg/mL (≈38 µM).

Recently, it was shown that the antiviral effect of potential antiviral compounds against SARS-CoV-2 can be cell-line-dependent. Notably, chloroquine blocked SARS-CoV-2 infection in Vero E6 cells, but it failed to neutralize SARS-CoV-2 infection in human Calu-3 cells [32]. This finding highlights the importance of using human cell line models such as the Calu-3 cells in addition to the commonly used Vero E6 cell line. We demonstrated that curcumin efficiently inhibited SARS-CoV-2 infection in both cell lines, Vero E6, and human Calu-3 lung cells, thereby indicating a genuine antiviral effect of curcumin against SARS-CoV-2.

In addition to the antiviral activity, curcumin also exhibits anti-inflammatory effects. Randomized controlled trials indicated a significant downregulation of the human tumor necrosis factor alpha (TNFα) and interleukin 6 (IL-6) through curcumin supplementation [33,34]. A meta-analysis showed that 8–12 weeks of treatment with 1 g curcumin per day can reduce symptoms of rheumatoid arthritis such as pain and symptoms related with inflammation [35]. However, no definitive conclusion can be drawn due to the small number of randomized controlled trials included in the analysis and small sample sizes. Furthermore, an add-on therapy with curcumin capsules improved airway obstruction in bronchial asthma patients [36]. Due to the antiviral as well as anti-inflammatory effect of curcumin, the compound might have a positive effect on COVID-19 progression. A clinical trial registered in Iran currently investigates the effect of curcumin–piperine co-supplementation on clinical symptoms, duration, severity, and inflammatory factors in patients with COVID-19 (IRCT20121216011763N46). Moreover, another clinical trial from Iran is studying the effect of curcumin-containing nanocarriers on symptoms of COVID-19 and inflammatory markers (IRCT20200611047735N1).

Turmeric root is generally recognized as safe (GRAS) by the U.S. Food and Drug Administration (FDA) [37]. Furthermore, the FDA concluded that when used as a flavoring agent or ingredient of specific foods, levels of up to 20 mg curcumin per serving are safe [38]. The European Food Safety Authority (EFSA) panel concluded that evidence supports an acceptable daily intake (ADI) of 3 mg/kg bodyweight per day for curcumin [39]. A clinical trial indicated that curcumin is not toxic for humans when administered at doses ranging from 1 to 8 g/day for up to 3 months [40]. In addition, other human trials using 1–2 g/day also reported curcumin as safe [41]. When administered at high doses of up to 12 g/day, only mild side effects such as diarrhea, headache, rash, and yellow stool were reported [42].

The clinical use of curcumin is hindered by its poor bioavailability. Only 1% of curcumin is absorbed by the body, and after a half-life of approximately 8 h, it degrades into several ineffective products [43,44,45,46]. Methods, involving the use of nanoparticles, liposomes, micelles, and adjuvants should be used to enhance the bioavailability of curcumin [47,48]. For instance, the bioavailability of curcumin can be increased by 2000% when using piperine as an adjuvant [47]. Further studies are required to determine the dose needed to reach adequate serum and lung tissue concentrations sufficient for virus neutralization.

Taken together, we demonstrated that curcumin potently neutralizes SARS-CoV-2 in vitro at low subtoxic concentrations. The good safety profile of curcumin and its immunomodulatory as well as the antiviral effect make curcumin a promising candidate for complementary treatment of COVID-19. Clinical studies evaluating the benefit of curcumin treatment in COVID-19 patients are pending.

## Data Availability

The data presented in this study are available on request from the corresponding author.
